# Social comorbidities? A qualitative study mapping syndemic theory onto gender-based violence and co-occurring social phenomena among Brazilian women

**DOI:** 10.1186/s12889-020-09352-7

**Published:** 2020-08-18

**Authors:** Casey D. Xavier Hall, Dabney P. Evans

**Affiliations:** 1 Medical Social Sciences, Feinberg School of Medicine, Northwestern University, Chicago, IL USA; 2 Institute for Sexual and Gender Minority Health and Wellbeing, Northwestern University, 625 N. Michigan Avenue, Suite 1400, Chicago, IL 60611 USA; 3 Hubert Department of Global Health, Rollins School of Public Health, Emory University, Atlanta, GA USA

**Keywords:** Brazil, Gender-based violence, Femicide, Intimate partner violence, Adverse childhood experiences, Family violence, Community violence, Violence, Substance use, Syndemic

## Abstract

**Background:**

Gender-based violence is a globally recognized social problem impacting women and girls worldwide. Intimate partner violence (IPV) represents the most common form of gender-based violence. Among the countries grappling with gender-based violence is Brazil, which has identified high rates of IPV along with co-occurring social conditions such as adverse childhood experiences, community violence, and substance use. While the syndemic framework has incorporated IPV into understandings of HIV and other diseases, none have explicitly applied syndemic framework to understand IPV and co-occurring social conditions -- referred to here as “social comorbidities” -- in the absence of a biological outcome. This study aims to: (1) Examine perspectives on violence and relevant social comorbidities (substance use, community violence, and childhood abuse) among women living in Santo André, São Paulo State, Brazil; and (2) Apply the syndemic framework to a set of social comorbidities among women living in Santo André, São Paulo State, Brazil.

**Methods:**

This thematic analysis applies a syndemic framework to 28 in-depth interviews with women in Santo André, Brazil. Interviews were recorded and transcribed verbatim in Portuguese. Our analysis examined themes relating to IPV, community violence, substance use, and other individual experiences and community issues using syndemics as an organizing framework (e.g. diseases, adverse interactions, disparity conditions, and enhanced disease transmission).

**Results:**

Most participants described experiencing multiple social comorbidities including IPV, adverse childhood experiences, community violence, family violence, and substance use. Adverse interactions included increased financial conflicts, a sense of isolation, and increased severity of violence due to substance use. Long term enhanced “disease” progression included injury, increased mental health symptoms, femicide, and death.

**Conclusions:**

Our results suggest that using a syndemic framework to understand IPV in the context of social comorbidities could be useful for understanding how these social phenomena may mutually reinforce each other and cause adverse interactions. Similar applications across other social phenomena may also be possible.

## Background

Gender-based violence (GBV) is a globally recognized social problem [1]. GBV encompasses many types of violence that impact women and girls including, but not limited to childhood abuse, sexual assault, intimate partner violence (IPV), and femicide. IPV is the most common form of GBV with an estimated 30.0% of women aged 15 or older experiencing physical or sexual violence by an intimate partner in their lifetime worldwide [1]. Brazil — in addition to having high rates of IPV — faces elevated rates of non-lethal community violence, drug possession crimes, and homicide [2–5]. In this analysis, we examine this range of comorbid social conditions through a thematic analysis of qualitative interviews from Brazil conceptually framed with syndemics.

### Syndemic framework and IPV

Syndemics is defined as, ‘the aggregate of two or more diseases or other health conditions in a population in which there is some level of deleterious biological or behaviour interface that exacerbates the negative health effects of any or all of the diseases involved’ (p. 941). These interactions of clustering health conditions lead to adverse interactions, increased exposure, and exacerbated long term consequences [6]. Syndemic theory also suggests that these clustering health concerns are more likely to happen under conditions of inequality, poverty, stigma, stress, and structural violence [6]. Syndemics is a theoretical framework which has been used to examine the relationship between many health outcomes, including IPV [6]. Conceptualizations of syndemics encompass social and biological health phenomena, though it has most frequently been employed to examine infectious disease. Violence has long been considered a component of syndemics, in fact it was part of the first conceptualized syndemic in combination with HIV and substance use in what was known as SAVA (substance abuse, violence, and AIDS) [7]. Similarly, in the violence literature IPV has long been examined in relationship to other social or behavioural health concerns. Drawing upon both fields we introduce a new terminology for referring to co-occurring social phenomena when applying the syndemics framework which we refer to as social comorbidities. To our knowledge our paper is the first to explicitly apply the syndemic framework to understand IPV and co-occurring social conditions in the absence of a biological outcome.

### IPV and social comorbidities

IPV is correlated with a range of social phenomena including adverse childhood experiences, community violence, sexual assault, and substance abuse [8]. Moreover, IPV has been linked to several of these outcomes in analyses addressing syndemics, but are generally connected to a central biological outcome or infectious disease such as HIV [6]. However, the syndemic framework has been examined far less in the absence of a central biological outcome or infectious disease – and even less frequently with a behavioural phenomenon as the focus. While syndemic frameworks explicitly encompass biological, behavioural, and sociological phenomena, the incorporation of syndemic frameworks has largely centred biological outcomes which is underlined by the biological framing of the syndemic framework in terminology such as ‘disease’ to refer to the syndemic social and biological conditions [6]. Yet, social conditions are time and time again associated with IPV [8, 9].

When applying the syndemic framework in the absence of a biological outcome, we propose considering these social conditions as *social comorbidities*, which we define as the social and/or behavioural phenomena that often (though not always) accompany IPV as antecedents and co-occurring phenomena. Possible social comorbidities include childhood sexual abuse, family violence, community violence, sexual assault, depression, and substance abuse [8, 9]. These social phenomena are frequently examined as antecedents, risk factors correlates, or outcomes of IPV [8, 9]. The potential benefit of conceptualizing these phenomena as social comorbidities, is the recognition that if unaddressed they may co-occur in a way that promotes adverse reactions and intensifies long term health consequences such as femicide, the intentional murder of women, which accounts for approximately 60,000 deaths annually worldwide [10, 11].

### The context of Santo Andre, Brazil

Brazil, a country of 209 million in South America has faced considerable challenges in regard to GBV including ranking 5th among 83 countries for femicide with rates of 4.8 femicides per 100,000 women [12, 13]. The majority of these femicides are attributed to IPV [13]. Estimates of physical and sexual IPV in Brazil are as high as 36.9% [14] and psychological IPV estimates are between 41.8-48.9% [2, 3]. Beyond IPV, social comorbidities are also prevalent in Brazil. Generalized violence is an increasing concern including the overall homicide rate, which has been rising over the last 3 decades [4, 15]. In 2009, Brazil had an estimated 19 attempted homicides, 340 assaults, and 29 crimes of drug possession per 100,000 people based on crime data [4]. In 2008, Brazil held the 13th highest homicide rate in the world at 29.6 per 100,000 people compared to the world estimate of 7.9 per 100,000 worldwide [4]. This trend appears to be increasing in Brazil overall; In 2017 Brazil had an estimated 31.6 deaths per 100,000 [15]. Gender plays a role in this violence with the majority of aggressors of all forms of violence in Brazil being men (72%) [5]. These concerns have been met with a federal response that seeks to protect women and girls through federal laws such as the Maria da Penha law, which aims to reduce IPV through increased penalties and provision of protective measures [16].

São Paulo state in Southeastern Brazil is home to the most populated urban centre in the Americas. While homicide estimates in the Southeast of Brazil are lower relative to the rest of the country, they remain high compared to the rest of the world. In 2017 estimates were about 20 homicides per 100,000 people [15]. Femicide in São Paulo state has fluctuated in the last decade and in 2017 accounted for 2 homicides per 100,000 women in 2017 [15]. Moreover, São Paulo city has demonstrated high rates of IPV with estimated rates of physical or sexual IPV at approximately 28.9% [14]. Santo André is a suburb of Metropolitan São Paulo with a population of approximately 700,000 people and an economy that is primarily based in industry and manufacturing [17]. Santo André has faced a growing concern regarding violence with more than a 100% increase in violence against women cases between 2009 and 2013 [18]. To its credit, Santo André housed the only Municipal Secretariat for Women’s Policies (*Secretaria Municipal de Políticas para as Mulheres*) in the region from 2014 to 2016. The SPM was a government agency which aimed to formulate and implement prevention and intervention for gender inequality through policies and programs [19]. During its existence, the SPM fostered intersectoral collaboration addressing GBV, but in the wake of shifting federal administrations it was absorbed into the Department of Social Policies in 2017 [19].

### Current study

This paper applies constructs from syndemics as an organizing framework for the thematic analysis of qualitative interview data from women living in Santo André, São Paulo State, Brazil. The purpose of the study was to examine social comorbidities with IPV in the absence of a central biological health outcome with the following aims:

1. Examine perspectives on violence and relevant social comorbidities (substance use, community violence, and childhood abuse) among women living in Santo André, São Paulo State, Brazil; and
2. Apply the syndemic framework to a set of social comorbidities among women living in Santo André, São Paulo State, Brazil.

## Methods

This analysis was part of a larger study examining individual experiences and community perceptions of GBV. Detailed information on the methods of the parent study are available in earlier publications [20, 21]. The project was conducted in partnership with the SPM in Santo André, the Municipal Ministry of Health, and the ABC School of Medicine (*Faculdade de Medicina do ABC*).

### Data collection

Thirty in-depth interviews (IDIs) were conducted by a native Portuguese speaker in 2016. Participants were recruited from public health clinics in three neighbourhoods of varying socio-economic status. Participants identified as women, were 18 and older, and resided in Santo André. Using an original IDI guide, participants were asked open-ended questions about personal experiences and perceptions of GBV which have previously been described [20]. Follow-up questions, probing, and prompts were used to explore any experiences of violence such as interactions with law enforcement officials. The WHO protocol for conducting research on violence against women was applied throughout recruitment and data collection processes to ensure the safety of participants, clinic staff and researchers [22].

### Data management and analysis

All IDIs were audio recorded, transcribed verbatim in Portuguese, and de-identified. Data were coded and analysed for deductive and inductive themes using MAXQDA12 software (VERBI GmbH, Berlin, Germany). Two native speakers of English who are also fluent in Brazilian Portuguese applied codes from an English codebook allowing us to code in the original language. The researchers conducted a thematic analysis using deductive themes derived from the domains of inquiry; inductive themes emerged iteratively via close reading. Thematic codes were drafted and tested by multiple research staff including the second author. Drafts of the codebook were applied by 2 coders and revised based on observed discrepancies. Final coding was conducted by both coders and any remaining discrepancies were reconciled by one coder.

This analysis specifically examined the thematic codes of IPV, community problems, community violence, and alcohol and drugs. Participants were classified by the number of these themes that they discussed. Participants that did not discuss any of these themes (*n* = 2) were excluded from this analysis. The remaining participants (*n* = 28) discussed at least one of these themes during the course of their interview. The relationships between codes were examined within participant, and then compared across participants. Observations were then categorized under constructs from the syndemic framework (diseases, adverse interactions, disparity conditions, and accelerated transmission/progression). Only quotes which were included in the manuscript were translated into English by the first author and reviewed by the second author along with a native speaker of Brazilian Portuguese.

### Study ethics

The study protocol was reviewed and approved by Emory University’s Institutional Review Board, the Santo André municipal government, and *Plataforma Brasil*, the Brazilian national Institutional Review Board (CAAE 57344616.0.0000.5484). Personnel obtained written informed consent from all participants and assured participants that they could withdraw from the study at any time. All data were kept confidential and there was minimal risk associated with participation. At the end of the interview all participants received VAW-related materials including educational pamphlets and contact information for local resources.

## Results

Study participants lived in 14 neighbourhoods in municipal Santo André. Half of the sample self-identified as Afro-Brazilian, mixed race or another non-white identity (*n* = 15), and the remainder as white (*n* = 13). Participant ages ranged from 18 to 78, with a median age of 41.3. All but one lived with some form of family (partners, young children, and/or other relatives). About a quarter (*n* = 8) did not complete primary school. Most (*n* = 18) completed high school, and two had college-level instruction. Average monthly household income was about $360, but almost half (*n* = 12) lived in households earning under $270 per month. Most women (*n* = 18) were unemployed. Seventeen (*n* = 17) identified as Evangelical Christian while most of the remainder (*n* = 10) identified as Catholic. See demographics in Table 1.

**Table 1:** Demographic characteristics of participants (*n* = 28)

Variables	N	%	Mean	SD
**Neighborhood**				
Centreville	1	3.6		
Cidade São Jorge	9	32.1		
Jardim Santa Christina	3	10.7		
Jardim Santo Antonio de Padua	1	3.6		
Jardim Teles de Menezes	1	3.6		
Jardim, Santo André	2	7.1		
Parque Marajoara	2	7.1		
Parque Novo Oratório	1	3.6		
Principe de Gales	1	3.6		
Vila Sacadura Cabral	2	7.1		
Sítio das Vianas	1	3.6		
Vila Guiomar	2	7.1		
Vila Lucinda	1	3.6		
Vila Suiça	1	3.6		
**Age**			41.3	14.2
**Race**				
Black	6	21.4		
Multi-racial	7	25.0		
White	13	46.4		
Other	2	7.1		
**Education**				
None	1	3.6		
Primary School	6	21.4		
Some Secondary	9	32.1		
High School Diploma	10	35.7		
Some College	1	3.6		
College Degree	1	3.6		
**Religion**				
Catholic	10	35.7		
Evangelical	17	60.7		
None	1	3.6		
**Relationship Status**				
Single	7	25.0		
Separated	2	7.1		
Divorced	1	3.6		
Widowed	3	10.7		
Cohabitating	6	21.4		
Married	9	32.1		
**Family Monthly Income**			1297.7 Reais	1130.6 Reais
**Employment Status**				
Unemployed	16	57.1		
Unable to work/on leave	2	7.1		
Employed	8	28.6		
Retired	2	7.1		

Overall, participants discussed three main types of violence exposures: IPV, familial violence, and community violence. In addition to these forms of violence participants also discussed substance and alcohol use. Out of the 28 interviews only 5 (18%) women did not disclose a story about witnessing or experiencing violence, though they did discuss the themes included in the analysis. Almost half (*n* = 13, 46%) described experiencing IPV directly (physical, psychological, or sexual), a majority (*n* = 21, 75%) described witnessing some form of community violence with thirteen (46%) describing both experiencing IPV and witnessing some form of violence. It is important to note that experiencing, witnessing, and awareness of violence are recognized as indicators of trauma in the literature, including validated measures [23]. The participants described violent experiences, community violence, and substance use as being interrelated phenomena. Using syndemics as an organizing framework, we present the resulting analysis under the key constructs of syndemics: syndemic diseases, adverse interactions, enhanced disease progression, and disparity conditions [6].

### Syndemic ‘diseases’ or social comorbidities

Participants described several social comorbidities including experiencing and witnessing IPV, family violence, community violence, and substance use. Participants did not describe any related biomedical conditions or diseases with any regularity, so all comorbid conditions were of a social nature. Nearly all participants from the broader study (*n* = 28, 93%) discussed at least one social comorbidity and most of this subset (*n* = 20, 71%) spoke about at least three forms.

### Intimate partner violence

Participants described a range of experiences of IPV including mental/emotional, sexual, economic, and physical violence primarily perpetrated by boyfriends and husbands. Intimate partner violence was frequently attributed to jealous male partners, or insecurity in relationships around monogamy. Jealously was linked to actual cheating or the perception of cheating as shared by one participant,

*‘My eight year relationship was jealousy and betrayal, a lot of betrayal I didn't accept… he said ‘You want to separate from me for something that didn't happen,’ I said to him: ‘It didn't happen but I got it, I saw it.’ Then it started to drive him crazy there, he wanted to hit me ... he threw me on the floor and took a stick to hit me.’*

Abuse within marriages was also tied to relationship entanglement such as having children or sharing finances. Another participant described how she entered an abusive relationship after being raped and impregnated by her then boyfriend, later experiencing physical violence,

*‘When it happened, I thought, I thought, ‘now nobody will marry me anymore, because I'm not a virgin,’ and I'm sure even if my mother knew what happened, she was going to make me marry, because I got married, my whole life, almost 17 years of marriage, I kept getting beaten, kicked, drug out of the house by the hair, starving with my children, three sons…he worked, from 13 years old until 1996, he always worked, he just didn't like hard work, but the money that he took he spent everything with [another] woman. I starved, I went cold, still with three children.’*

This participant’s perceptions around virginity, and stigma surrounding sexual assault also underline the gendered dynamics of intimate partner violence, which were mirrored in other participants’ responses.

### Family/childhood violence

Participants described familial violence in a variety of ways including during childhood in their family of origin as well as later in life outside of IPV. Familial violence was described as both violence that the participant was directly engaged in as well as violence between other family members that the participants witnessed or heard of second hand. Though participants did describe adverse childhood experiences none of the participants disclosed childhood sexual abuse and we did not probe on this topic. Participants described directly witnessing violence during childhood such as seeing their father abuse their mother, or siblings that would get in physical fights. For example, one participant described her brother who was aggressive toward his mother and to anyone he came in contact with.

*‘He always was [aggressive], he always was… he lived at home, because he didn’t have a house to live in. My mother kicked him out of the house, because he can’t live near us. Everywhere he lives he gets into a fight and nobody can stay. If you rent him a place to stay, he breaks everything. He explodes wherever he goes… He broke [my mother’s] glasses and she was without glasses for the longest time.’*

Another example is witnessing the abuse of a mother at the hands of a father. For example, one participant described how her mother stayed in an abusive relationship for years, because she felt obligated to stay in the relationship.

*‘My father had another woman and he was a womanizer. He beat my mother a lot, but my mother thought she had to take this thing to the end.’*

Participants also recounted violent partners’ stories of witnessing violence. One participant described the experience of her ex-husband witnessing his father beat his mother and attempting to protect his mother.

*‘She was attacked… physically. My ex-husband… often witnessed [it]. He would fight his father to protect his mother… He would not accept his father beating his mother. That’s why I am telling you, he never assaulted me…’*

While this quote on its own may seem to denote a protective benefit of witnessing violence the same participant later shared that her partner verbally abused her when drinking.

*‘You know the person when they drink, their word is shameless, ‘slut’ ... They use it a lot.’*

In keeping with much of the IPV literature, the connection between witnessing abusive experiences during childhood and later perpetration of violence by males was substantiated. It also highlights how verbal abuse is not always characterized by participants in the same light as physical violence – or in the same categories as researchers.

Participants also described being abused by parents or other relatives as a child. One participant described her mother ordering her brothers to physically discipline her as a child. She described a difficult childhood, with strict gender roles where she was expected to do all of the house work. This same participant later described violence perpetrated by her brother as a form is ‘discipline’,

*‘My mother gave my brother, one of the oldest, the responsibility of ... of this way, of educating the younger ones, and this brother of mine, he would come to my house to beat us, but the younger ones would ask my mother if I was doing the right job, if I was obeying her, then he would go there hit us…it was like this ... a very difficult adolescence you know, very hard, childhood too, already working at home, doing everything, taking food in the fields, I have no good memory of my, me as a child, nor of my teenage years.’*

In keeping with the correlation between early exposure to family violence and the likelihood of female victimization, this same participant later described her prolonged experiences of intimate partner violence.

Many participants described experiencing both childhood abuse and intimate partner violence. However, direct experiences of violence as an adult also included violence perpetrated by family members other than a spouse. For example, one woman — who herself was a survivor of IPV — described the IPV her daughter experienced underscoring the intergenerational nature of IPV; the participant also described being attacked by her son-in-law who drank heavily,

*‘He drank and [my daughter] left. He was there in the house making a mess and breaking everything. Then he tried to punch me. My son came and told him that he couldn’t hit me. My son-in-law said ‘I can hit anyone.’ Then my son said ‘You can’t beat my mother’… and [my son] told him [my son-in-law] to never enter my house again. Now my son and I don’t talk to him.’*

Similar experiences were frequently discussed as connected to drugs, alcohol, and money. They were almost exclusively about male family members and included a range of familial ties such as brothers, sons, sons-in-law, or cousins.

### Community violence

Participants described witnessing, hearing about or being aware of violence in their communities. This type of violence ranged from public IPV to other forms of interpersonal violence such as physical fights in a public setting. Participants attributed public forms of violence to jealousy, insecurity, and finances as well as heavy drinking or substance use. These attributions aligned closely with how participants described their own experiences of violence. The vast majority of participants described witnessing community violence. One participant described an instance of public intimate partner violence:

*‘Oh, every now and then, right there I hear fights, husband shouting at wife. Who is right I don't know. I don't know the person, I just hear screaming.’*

Community violence was frequently connected to substance use such as drinking at a bar. For example, one participant described a nearby bar that was known for fights within her neighbourhood. Including fights between those drinking and couples.

*‘In the street down the road, there is a bar where people fight, sometimes because they are drunk. Sometimes it’s a couple, too.’*

Community violence also included descriptions of violence between police and people in the neighbourhood who committed crimes. One such example was one participant who described their ex-boyfriend being shot by police. This occurred when the police followed-up on a robbery the ex-boyfriend committed. This example is discussed further as a part of *enhanced disease transmission*.

### Substances

Alcohol and drugs were talked about similarly to each other in the interviews and very frequently linked to all three types of violence: IPV, family, and community violence. Alcohol was talked about much more frequently than drugs, though participants discussed both. Drinking was frequently framed as a reason for ‘different behaviour’ in a partner who is generally not violent.

*‘Then when he drinks he starts to treat me differently. My daughter says she doesn’t like it, a guy like him. ‘What does he do that’s useful? What does he offer?…Have you ever imagined the future? You’re going to live with this man in suspense. You don’t know if he is coming drunk.’*

A connection was also drawn between substances and other forms of violence such as familial violence and community violence. For example, one participant described how her father-in-law would drink heavily and verbally abuse her mother-in-law and get into fights with other people including the participant herself.

*‘When he left work at noon he would go straight to the bar. He would drink and when it was about 5 p.m. he would come down and when he got to the gate he would shout at my mother-in-law ‘you slut!’ My mother-in-law—the poor thing—was a saint… Because of his drinking he got into a lot of fights in all the places that he went.’*

Ultimately, in many cases participants linked drugs conceptually to violence and aggression of all kinds. In some cases, nearly all of the aggressors that participants described across their lives were men under the influence of a substance and/or alcohol.

### Disparity conditions

Disparity conditions were discussed primarily in the context of financial concerns. One participant suggested that most fights were about: *‘Money, lack of money, financial situations. I think that’s what gets the most people these days.’* Finances were often directly linked to drugs. One participant discussed this connection to money as well as employment status.

‘*My sons-in-law were drug users, now by the grace of god they are free of this problem. My husband drank, he had several addictions. They are gone now… Yes, [there are reasons such as] money, unemployment, everyone wants to raise their kids their own way. … unemployment is very difficult right.’*

Financial dependence was also a factor that was described as being connected to relationship entanglement such as the presence of children. *‘Because of the child, they have to be together.*’ However, financial concerns were not frequently discussed in the context of structural poverty or other macro conceptualizations; gender oppression, on the other hand was discussed on a macro-level.

Though gender oppression was typically more implicitly discussed, one participant, a survivor of violence, shared her idea of why childhood abuse experiences matter and framed IPV within a broader context of violence against women as a form of oppression.

*‘Ah, I think the meaning of violence against women, regardless of whether it is physical or moral, right, I believe it is a form of pressure [for] women right, to slow her down to prevent her from imposing herself on society. Often within your own home, within your own family. I think it's a form of oppression… I think nothing justifies [violence against women]. Although I understand that in these new relationships, which are very unprepared, very young, I think that violence often happens because of [the couples’] family history and unpreparedness. Because, usually, offenders, they have this family background; It's very hard for us to see an attacker who doesn't have a troubled track record.’*

While this critique was not explicitly stated by other participants, some other participants did allude to gendered dynamics of violence.

#### Adverse interactions

Adverse interactions between these social comorbidities were difficult to extract, because these comorbidities were frequently discussed in concert, rather than in isolation; this made discerning how each comorbidity functions in isolation difficult as compared to a combined effect. For instance, substances were often tied to violence, and financial irresponsibility. One participant who had a verbally abusive husband talked about how violence is linked to money and drugs,

*‘It's because of money right; so drinks cost money right and then fighting starts, and arguing with each other, the husband is there wanting to hit his wife right now, I was not born for that. Then the money is missing to pay rent, to pay things right, then they are preoccupied, and he wants to fight with her.’*

Financial tensions, conflict and drugs could be mutually exacerbating. Some participants described how multiple comorbidities contributed to a sense of isolation. One participant’s story illustrated how multiple comorbidities worked in concert in her life to contribute to this sense of isolation,

*‘My life is difficult, to this day I am alone, I have three children, but one there in Rio de Janeiro and two here, but each one lives their life, I am sick I have already starved inside my house. I found a month-old bread that was there right and ate it. I have to buy my food. I have to take myself to the doctor… I'm a very lonely person, very lonely… My family is big, my mother had 17 children…my father had with another woman, my father was very womanizing too, beat my mother a lot, but my mother thought she had to take this thing to the end… I have a scar here, see… My son broke a broom in my head… because I went to ask him for money for the utility bill.’*

#### Enhanced disease transmission

Conceptualization of enhanced ‘transmission’ or ‘progression’ was the most difficult construct to apply to social comorbidities perhaps due to the biological formulation of the construct. Additionally, data on increased progression was sparse in these data; however, participants did discuss more advanced forms of victimization that lead to physical injury or death. For example, one participant discussed being assaulted by a jealous partner and how drinking was involved.

*‘Sometimes we would fight, but once he assaulted me. I even took a stab here [gestures to body]. I almost died. But then again, I fought back. So, I stayed. It was about jealousy. ‘*

Several participants also spoke the effects of violence on mental health. One specifically stated that the emotional scars were worse than the physical ones. *‘I am not talking about physical health. I am talking about emotional health. The marks on your body fade, but the [emotional] stay.’*

Participants did discuss perceptions of things like murder, femicide, and death. Several participants discussed how partners who were perpetrators of violence died under circumstances related to aggression, or under unclear circumstances. For example, one participant described a perpetrator who was run over and killed by a car, which she perceived as possibly intentional,

*‘He was jealous of her… She suffered, but I think she loved him, and they had children, everything. Today, her daughter got married. Her son was killed by the police. Her husband also died ran over by a car, but I think they really wanted to kill him.’*

Another described how her ex-husband’s propensity for fighting led to his death in a public brawl.

*‘He started drinking and staying that way. Only then we separated. I got a good break, but he died later, because he fought with his cousin. They argued… when it was over, his cousin hit his head and broke his neck.’*

Participants also mentioned stories of people who killed by police, for one participant this included her boyfriend who had been a drug dealer and was ultimately shot when fleeing the police.

‘*This guy I dated for 3 years. When I was 16 I found out he had two jobs. By day he was a mechanic and at night he was a drug dealer. It was that side job that I didn’t know… One day when I came home from school, I passed by his house to get to mine, and he came and took my arm: ‘let me talk to you,’ he said. I said: ‘I don’t want to talk to you leave me alone it’s over, we’re not getting back together’…That same day at dawn he committed a robbery—Look at what I was getting myself into—the police were notified and they told him to reveal who had told him to commit the robbery and he didn’t want to talk. He said, ‘I’ll die, but I don’t care.’ He ran from the police and they shot 23 times… That’s when my depression began. I discovered that I had bipolar disorder a little while later, but my depression began at 13. My experience with [sexual] violence was at 18.’*

While these examples were focused on the death of violent perpetrators, participants also discussed the salient concern of femicide. This came up in cases where women report violent partners, but feared retaliation.

*‘I saw on the television a commercial that spoke about the Maria da Penha law, but I don’t have faith in it, because they get out. Women remain afraid and trapped. Yesterday there was a case where a woman lost her job because she feared leaving her house to be killed.’*

These fears are reinforced by stories about women who were killed by their partners or whose partner attempted to kill them after reporting violence to officials.

*‘I know of a case of a woman who now lives today in S*ã*o Paulo (city), her husband stayed here because he threatened to kill her. He went to the door of the school where she works to kill her, and she had to run away with her two year old daughter.’*

These stories reinforce that aggravated forms of prolonged violence can result in very serious consequences for the women involved including death.

## Discussion

**Fig. 1:**
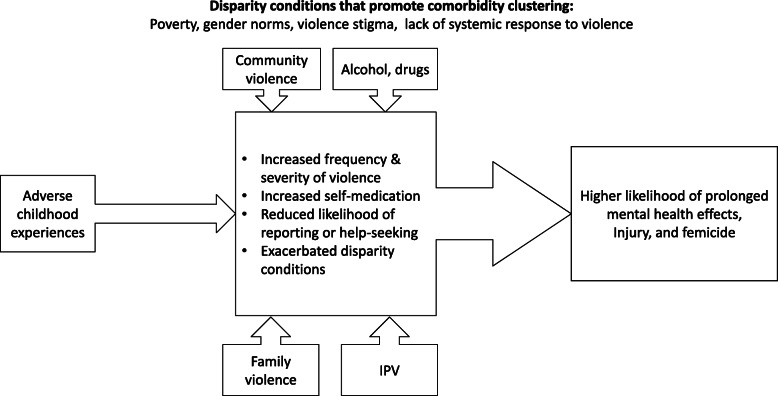
A Conceptual Model of the Syndemic Social Comorbidities of Intimate Partner Violence in Santo André, Brazil

This analysis illustrates how a syndemic framework can be applied to a set of social comorbidities in the absence of a biological outcome. In this case, IPV, family violence, community violence, and substance abuse can be understood as syndemic social comorbidities in the sense that they are mutually re-enforcing social phenomena (see Fig. 1). However, the language of syndemics may need adaptation when applied to only social phenomena, because syndemics are largely framed in a biological manner. In our analysis, we saw that: 1) syndemic social comorbidities of IPV, familial violence, community violence and substance use were described as interrelated health concerns; 2) some of these comorbidities were discussed across the lifespan and including adverse childhood experiences; and 3) these interrelated concerns led to adverse interactions and enhanced progression such as depression and even femicide. Understanding gender-based violence in the context of syndemic social comorbidities may inform the future study of these phenomena including the development and implementation of integrated approaches to intervention.

### Evidence for syndemic social comorbidities

This analysis aligns with previous understandings of IPV from the literature in a number of ways. For instance, participants described drugs and alcohol as factors contributing to their experiences of all forms of violence which is consistent with previous literature [8]. Indeed, in studies based in São Paulo alcohol use was predictive of violence against women [24]. Additionally, direct experience and secondary exposure to community violence may be linked in part to IPV through generally aggressive partners. Women in São Paulo who have partners who fight with other men are more likely to experience violence [25]. Similarly, the construct of *disparity conditions* was discussed in regard to personal financial struggles, which aligns with literature stating that unemployment and lower income have been linked to IPV [8]. Moreover, sexual violence has been found to be more common in areas with lower education, lower income, and higher unemployment in Brazil [26]. Participants described gendered patterns of violence and one participant explicitly articulated the role of sexism in gender-based violence, which suggests that underlying sexism may be considered a *disparity condition* in syndemic models. Ultimately, participants described *adverse interactions* and long-term consequences of IPV and comorbid factors highlighting mental health symptoms, injury, and even femicide. Both direct experience of violence and indirect experiences of violence have been linked to psychological distress in Brazilian samples [27] and the majority of femicides in Brazil are attributed to IPV [13].

### Life course exposures and syndemic social comorbidities

This analysis also raises questions about social phenomena which occur earlier in life, or over multiple developmental periods and how they should be conceptualized in a syndemic social comorbidity framework. Most notably, this study linked experiencing or witnessing abuse as a child to the experience of IPV later in life. This is consistent with previous literature addressing predictors of IPV [8]. In Brazil, adverse childhood experiences are common. As many as 60% of children in one study witnessed violence against their mothers as children [28]. Women in Brazil are more likely to have experienced childhood abuse than men [29], which highlights one way adverse childhood experiences overlap with gender-based violence more broadly. Despite its relevance, the conceptualization of adverse childhood experiences are difficult to place in a syndemic framework. This kind of social phenomena does not neatly fit into the construct of a syndemic ‘disease,’ because the experience itself may have occurred long before experience of IPV, though the effects of childhood experiences may persist throughout life [6]. Additionally, childhood abuse does not align clearly with the construct of ‘disparity conditions,’ because it is not necessarily a contextual condition nor is it directly linked to disparity [6]. Future conceptualizations of social comorbidities—or syndemics more broadly—may benefit from the addition of a childhood exposures construct.

### Centring social phenomena

By centring social phenomena in the syndemic framework we problematize the emphasis on biological conditions such as infectious diseases that is inherent in the framework. Syndemic frameworks have made significant contributions by integrating co-occurring biological, social, and behavioural health concerns under a single framework that recognizes the mutual reinforcing and adverse interactions of these co-occurring conditions [6]. Our conceptualization of social comorbidities pushes the boundaries of the framework to encompass health-related phenomena that are exclusively social and behavioural in nature. This analysis highlights the dissonance of the constructs used in relation to social and behavioural phenomena as they are conceptualized as *diseases* in applications of syndemics [6]. The most notable conceptual incongruence in this analysis arose when considering the concept of accelerated *transmission* whose social analogue may be *diffusion* or alternately *social norms* which is more in line with conceptualizations of violence as a social contagion [30].

### Broadening an understanding of disparity conditions

While syndemic models are conceptualized within the context of disparity conditions, the framework does not fully articulate these conditions beyond their role in promoting disease clustering [6]. Thus, study of syndemics or social comorbidities could benefit from the integration of the social determinants of health framework as well as social-ecologic models [31, 32]. Disparity conditions may refer to social environment, material circumstances, the impact of social inequalities, and the trans-generational transference of these inequitable conditions [31, 33]. An understanding of key social determinants such as poverty or gender inequity may help inform social comorbidities, particularly to the extent that they are produced by these determinants [32]. Additionally, multi-level influences are not explicitly addressed within the syndemic framework; however, this may be addressed through efforts to define multiple levels of disparity conditions such as in social-ecological frameworks [34, 35]. Unlike the social ecological and social determinants of health frameworks, the syndemic framework does clearly define the relationships between key constructs including mutually re-enforcing health conditions, while social determinants address common antecedents and social ecological frameworks simply allow for recursive influences between levels of the social ecology [34, 35]. Thus, the integration of these frameworks may allow for the identification of multi-level factors that influence social comorbidities while examining the more clearly defined relationships between constructs in syndemic theory [6, 35]. This may also assist in the identification of integrative interventions at higher levels of the social ecology in order to reduce social comorbidities [35]. This could be particularly useful, because a focus on social phenomena implies intervention on higher levels of the social ecology rather than biological or individual intervention [35]. Future research should seek to better understand the impact of disparity conditions on social comorbidities, particularly potential interventions that address these conditions. In the context of Brazil, violence-specific policies such as the Maria da Penha law have seen some limited success; however, the integration of these conceptual frameworks may lead one to examine broader-reaching policies that address social determinants that are linked to social comorbidities such as poverty and gender-based inequity [2, 20, 21].

### Benefits to a social syndemic framing of IPV

Conceptualizing IPV and its co-occurring social phenomena as syndemic social co-morbidities is beneficial to the understanding of IPV for several reasons. First, it situates social phenomena in parallel. This allows researchers to understand social comorbidities as mutually influencing and to identify the potential for adverse interactions. This diverges from research that situates co-morbidities as causative and moves toward examining mechanisms through which the consequences of these social conditions may be intensified. Second, the conceptualization of adverse interactions and accelerated progression may be informative to our understanding of femicide and the conditions in which femicide may be more likely. Indeed, some comorbidities are already recognized as possible indicators of risk for femicide such as illicit drug use or particular types of physical violence such as choking [36]. Lastly, an understanding of syndemic comorbidity may influence approaches to prevention and intervention. Interventions that target syndemics may include initiatives that attempt to alleviate disparity conditions or that attempt to intervene on disease clusters and/or adverse interactions of disease clusters [6]. The most promising research emerging around IPV prevention supports the idea that multi-level interventions are the most effective way to prevent IPV incidence and reoccurrence. Interventions that address disparity conditions may incorporate financial support such as conditional cash transfer programs or micro-finance programs aimed at reducing poverty and encouraging positive health behaviours [37–40]. Some financial programs integrate an understanding of disparity conditions, and gender roles in relation to IPV and other forms of gender-based violence [40]. These programs also have potential for having impact on substance use such as in the case with the Stepping Stones program which saw reductions in substance use among men [40]. Integrative approaches may attempt to reduce multiple comorbidities through a single integrated program or through complementary coordinated programs [41]. Women who participated in the integrated program saw reductions in violence, increases in violence self-efficacy, and reductions in substance use [42]. Additional interventions that explicitly seek to address a fuller complement of social-comorbidities may be created and tested.

### Limitations

As a small qualitative study it is not possible to generalize the findings of this analysis to populations; though some of the lessons learned may be transferable to different contexts. The interview instrument was not designed to elicit syndemic constructs, and syndemics was used as an organizing framework for the qualitative analysis. This means that we were not able to probe on syndemics concepts and may not have reached saturation in all of the themes discussed. Future research on IPV may prospectively apply the syndemic framework in order to more adequately explore the advantages and disadvantages of such framing.

## Conclusion

Conceptualizing IPV in the context of syndemic social comorbidities is an informative framing for a critical health problem that has implications for women and girls in Brazil and globally. By adapting the syndemic framework to centre social phenomena, we are able to better examine the ways in which IPV, family violence, community violence and substance use interact and exacerbate each other. Those using syndemic models should consider ways to integrate early life exposures such as adverse childhood events which likely influence syndemic diseases and social comorbidities. Additionally, future studies should examine syndemic clustering of social comorbidities with intimate partner violence, especially in quantitative, longitudinal studies to further elucidate the ways that IPV, family violence, community violence and substance use exposure trajectories influence each other over time. Public health practitioners should also consider multi-level and integrated interventions to address social comorbidities.

## Data Availability

The datasets generated and/or analysed during the current study are not publicly available to protect the privacy of participants.

## Abbreviations

GBV: Gender-based violence
IPV: Intimate Partner Violence
